# Temporary Anchorage Devices in Clear Aligner Therapy: A Systematic Review

**DOI:** 10.3390/bioengineering12050531

**Published:** 2025-05-15

**Authors:** Grazia Marinelli, Angelo Michele Inchingolo, Alessio Danilo Inchingolo, Laura Ferrante, Pasquale Avantario, Merigrazia Campanelli, Andrea Palermo, Francesco Inchingolo, Gianna Dipalma

**Affiliations:** 1 Interdisciplinary Department of Medicine, University of Bari “Aldo Moro”, 70124 Bari, Italy; graziamarinelli@live.it (G.M.); alessiodanilo.inchingolo@uniba.it (A.D.I.); lauraferrante79@virgilio.it (L.F.); avantario@libero.it (P.A.); merigrazia.92@hotmail.it (M.C.); gianna.dipalma@uniba.it (G.D.); 2 Department of Experimental Medicine, University of Salento, 73100 Lecce, Italy; andrea.palermo@unisalento.it

**Keywords:** aligners, clear aligners, transparent aligners, Invisalign, temporary anchorage devices, skeletal anchorage, miniscrews, orthodontic anchorage systems, miniscrew implants

## Abstract

This systematic review analyzed the combined use of aligners and orthodontic temporary anchorage devices (TADs) in orthodontic treatment. The aim was to evaluate the effectiveness, benefits, and potential challenges of integrating the use of miniscrews with aligners. This review was conducted according to the PRISMA statement, and the protocol was registered at PROSPERO under the ID CRD42024576712. A comprehensive search on PubMed, Scopus, and Web of Science was conducted to identify relevant papers involving patients treated with aligners and TADs, dating from 1 January 2004 to 17 July 2024. The electronic database search identified a total of 458 articles. After eligibility, 14 records were selected for qualitative analysis. The findings suggest that the combination of aligners and miniscrews significantly enhances treatment precision and control, especially in cases requiring complex tooth movements, such as intrusion, extrusion, and distalization. The use of miniscrews allows greater control of movement and stability. The integration of these two techniques presents challenges, such as the need for precise miniscrew placement and potential discomfort during insertion. However, there was high satisfaction due to the aesthetic and comfort benefits of aligners. Further research is desirable to delve deeper into the topic to optimize clinical outcomes.

## 1. Introduction

### 1.1. Evolution of Orthodontic Treatment

Orthodontic treatment modalities have undergone significant advancements over the past few decades, providing patients and clinicians with a variety of options for correcting malocclusions and other dental irregularities. Traditionally, fixed appliances, comprising brackets, wires, and bands, have been the gold standard in orthodontic care [1,2,3].

### 1.2. Emergence and Workflow of Clear Aligners

In recent years, clear aligners have emerged as a popular alternative to fixed appliances, particularly among adult patients seeking more discreet treatment options. Made from transparent, medical-grade plastic, aligners are custom-designed from a digital impression of the patient to gradually move teeth into their desired positions [4,5,6]. Digital impressions are obtained using advanced intraoral scanners, capturing a detailed 3D representation of the patient’s dentition and occlusion. This process not only enhances accuracy but also significantly improves patient comfort and reduces chair time [7,8,9,10,11]. Following the digital scan, the data are processed to create a virtual model of the patient’s teeth, which is then used in ClinCheck Pro 5.0 software, a pivotal tool in aligner therapy. ClinCheck allows orthodontists to design a comprehensive, step-by-step treatment plan, visualizing tooth movements over time. The software provides a preview of the expected outcomes, enabling both the clinician and the patient to review and adjust the treatment [12,13,14,15]. Clear aligners have revolutionized orthodontic treatment, offering patients a more aesthetically pleasing and comfortable alternative to traditional braces [16,17,18,19].

### 1.3. Limitations of Aligners and the Role of TADs

However, while aligners provide numerous benefits, including enhanced hygiene and reduced treatment discomfort, they often face limitations in controlling complex tooth movements, particularly in cases requiring significant anchorage [20]. To overcome these challenges, integrating temporary anchorage devices (TADs) with aligner therapy has gained increasing attention in recent years [21].

### 1.4. Historical Background and Advances in TADs

TADs, first introduced in the early 2000s, represented a major shift in orthodontics. Early reports by Kanomi (1997) and Costa et al. (1998) described the use of small titanium screws inserted into bone to serve as anchorage points. These early TADs offered a novel solution for cases where traditional anchorage methods were insufficient. Over time, their application has expanded significantly, with newer studies highlighting improved placement techniques, stability, and compatibility with digital treatment planning systems.

### 1.5. Biomechanical Benefits of TADs

**Figure 1:**
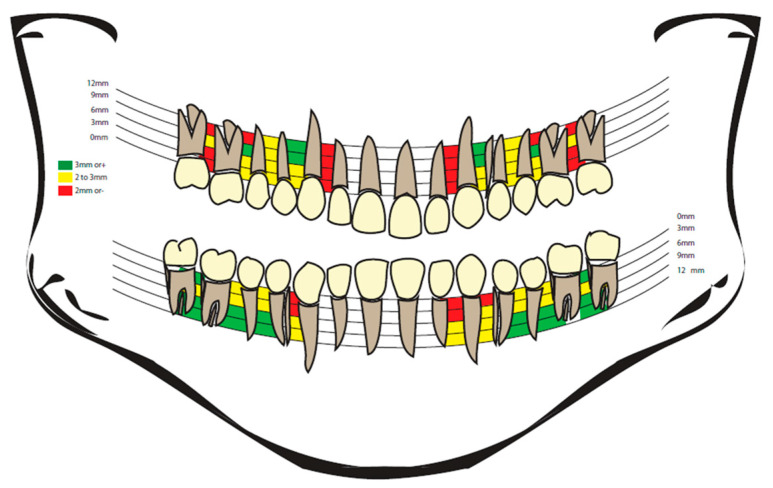
The position for insertion of TADs.

TADs have become a game-changing instrument, providing solutions to problems in attaining accurate tooth motions, especially when utilizing clear aligners [22]. Tiny titanium screws, often known as mini-implants or TADs, are placed temporarily into the palatal or alveolar bone (Figure 1) [23,24,25].

Their main purpose is to provide a solid anchorage point so that regulated forces may be applied to move teeth without causing the unwanted reciprocal movements that are frequently connected to conventional anchorage techniques. Although aligners work well for general teeth alignment, there are situations when they are not biomechanically efficient enough to carry out these motions precisely. TADs give aligners the essential anchoring to increase their effectiveness by enabling the application of extra forces in particular directions that aligners are unable to achieve by themselves [26,27,28,29].

### 1.6. Clinical Use and Indications

**Figure 2:**
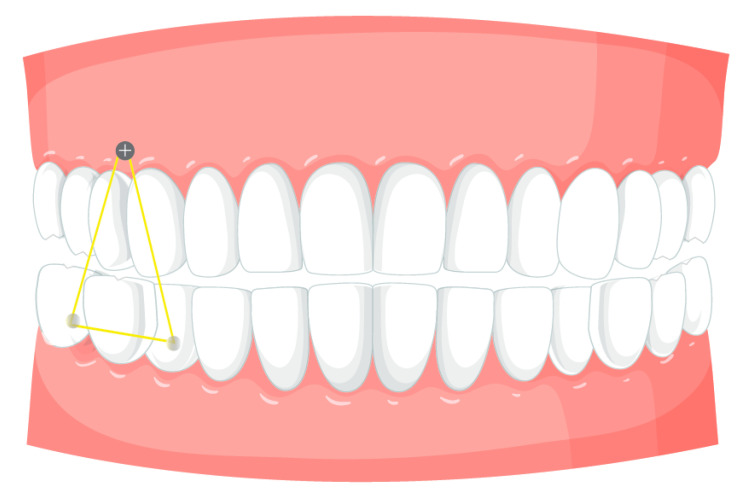
Extrusion of the lower posterior sector. The inter-arch elastic traction between the upper and lower arches is illustrated in yellow.

TAD implantation is a minimally invasive treatment that is usually carried out under local anesthetic. Choosing the right location, making sure there is enough bone density, and inserting the TAD with a specific driver are the steps in the procedure. Once installed, TADs can be employed to facilitate complex movements in combination with coil springs, elastics, or direct attachment to the aligners. TADs are simply removed once the intended orthodontic outcomes are attained, leaving little to no scarring or harm to the surrounding tissues [30,31,32,33,34]. In difficult instances where significant tooth movement is necessary, such as intrusion, extrusion, molar distalization, and correction of open bites or deep bites, the use of TADs in aligner-assisted orthodontic therapy is especially beneficial. TADs offer a stable anchorage point that can enhance the efficiency and effectiveness of orthodontic treatments (Figure 2) (Table 1) [35,36].

### 1.7. Combined Use of Aligners and TADs

The combination of aligners and TADs presents a promising approach for managing challenging malocclusions without the need for procedures that require the cooperation of the patient, which is crucial when using aligners [37,38]. This dual approach expands the range of cases that can be treated with aligners. Furthermore, the use of TADs in conjunction with aligners can significantly shorten treatment times and improve outcomes in complex cases, offering a minimally invasive solution that enhances patient satisfaction and compliance [39,40,41].

### 1.8. Recent Advances in Antimicrobial Coatings for Orthodontic Appliances

In the last few years, increasing attention has been directed toward the development of antimicrobial coatings for orthodontic appliances. These materials aim to reduce bacterial adhesion and biofilm formation, which are critical factors in maintaining oral health during orthodontic treatment. Recent publications have discussed the progress made in dental materials, highlighting the potential of incorporating antimicrobial agents, such as silver nanoparticles, chitosan, and quaternary ammonium compounds, into orthodontic appliances and aligners [42,43,44]. These innovations not only contribute to improved oral hygiene but also complement the use of clear aligners and TADs by reducing the risk of localized infection and inflammation around anchorage sites.

### 1.9. Comparison with Previous Systematic Reviews

While several systematic reviews have addressed either clear aligner therapy or the use of TADs independently, only a few have focused specifically on their combined application. Previous reviews have primarily examined clinical outcomes related to clear aligners or skeletal anchorage separately [20,45]. However, this review provides a comprehensive synthesis of the literature, focusing on their synergistic use. By evaluating not only clinical effectiveness but also patient-centered outcomes and biomechanical advantages, our study offers a broader and more updated perspective. Furthermore, it includes recent developments in digital planning, material science, and surgical protocols that were not addressed in earlier reviews.

### 1.10. Aim of the Review

This systematic review aims to evaluate the current literature on the combined use of aligners and TADs in orthodontic treatment, focusing on clinical outcomes, effectiveness, and potential complications [21,46,47]. The goal is to highlight the advantages and best practices associated with this approach, ultimately contributing to more predictable and successful orthodontic outcomes [48,49].

## 2. Materials and Methods

### 2.1. Protocol and Registration

This systematic review followed the guidelines outlined by the Preferred Reporting Items for Systematic Reviews and Meta-Analyses (PRISMA) [50]. The review protocol was registered at PROSPERO under the ID CRD42024576712.

### 2.2. Search Processing

A search on PubMed, Scopus, and Web of Science was performed to find papers that matched the topic of orthodontic treatment performed with aligners and TADs in patients requiring orthodontic treatment, dating from 1 January 2004 to 17 July 2024, in English. The search strategy used the following Boolean keywords:

(aligners OR clear aligners OR transparent aligners OR Invisalign) AND (Temporary Anchorage Devices OR TADs OR OrthoTADs OR Mini-implants in orthodontics OR Skeletal anchorage OR Mini-screws OR Micro-implants orthodontics OR Orthodontic anchorage systems OR Miniscrew implants OR Temporary skeletal anchorage devices OR Orthodontic biomechanics with TADs) (Table 2).

### 2.3. Inclusion Criteria

The following inclusion criteria (Table 3) were considered:

(1) Studies involving patients undergoing orthodontic treatment with both clear aligners and Temporary Anchorage Devices (TADs);
(2) Studies that specifically addressed malocclusions requiring complex tooth movements, such as Class II, Class III, anterior open bite, deep bite, and molar distalization;
(3) Studies involving human participants aged 12 years and above, including adolescents and adults, with no craniofacial syndromes or systemic diseases;
(4) Randomized clinical trials, retrospective studies, case series, and case reports;
(5) Full-text articles published in English.

Papers not fulfilling these criteria were excluded.

The PICOS strategy was applied as follows:

- Participants: Male and female patients aged ≥12 years, requiring orthodontic treatment with no syndromic or systemic pathology.
- Intervention: Orthodontic therapy combining clear aligners and TADs.
- Comparison: Treatment outcomes before and after intervention.
- Outcome: Evaluating the efficacy of clear aligners with TADs in treating complex malocclusions.
- Study Design: RCTs, retrospective studies, case series, and case reports.

**Table 3:** Inclusion and Exclusion Criteria.

Category	Criteria
Inclusion	Clinical studies (RCTs, retrospective studies, case series, case reports) Orthodontic treatment with aligners and TADs English language Full-text available
Exclusion	Animal studies In vitro studies Off-topic articles Reviews, letters, comments Non-English language articles

### 2.4. Exclusion Criteria

The exclusion criteria were as follows: (1) animal studies; (2) in vitro studies; (3) off-topic; (4) reviews, letters, or comments; (5) non-English language.

### 2.5. Data Processing

Three reviewers (G.M, A.D.I., and G.D.) independently consulted the databases to collect the studies and rated their quality based on the selection criteria. The selected articles were downloaded into Zotero (version 6.0.15). Any disagreement among the three authors was resolved through discussion with a senior reviewer (F.I.).

### 2.6. Quality Assessment

The quality of the included studies was independently assessed by two reviewers (M.C. and P.A.) using the ROBINS-I tool, which is designed to evaluate the risk of bias in non-randomized studies comparing the health effects of two or more interventions. Seven domains were assessed, each rated for potential bias. In cases of disagreement, a third reviewer (F.I.) was consulted until consensus was achieved.

The questions in the domains evaluated in ROBINS-I are as follows:

- Bias due to confounding;
- Bias arising from the measurement of the exposure;
- Bias in the selection of participants in the study;
- Bias due to post-exposure intervention;
- Bias due to missing data;
- Bias arising from the measurement of the outcome;
- Bias in the selection of the reported results.

## 3. Results

### 3.1. Study Selection and Characteristics

**Figure 3:**
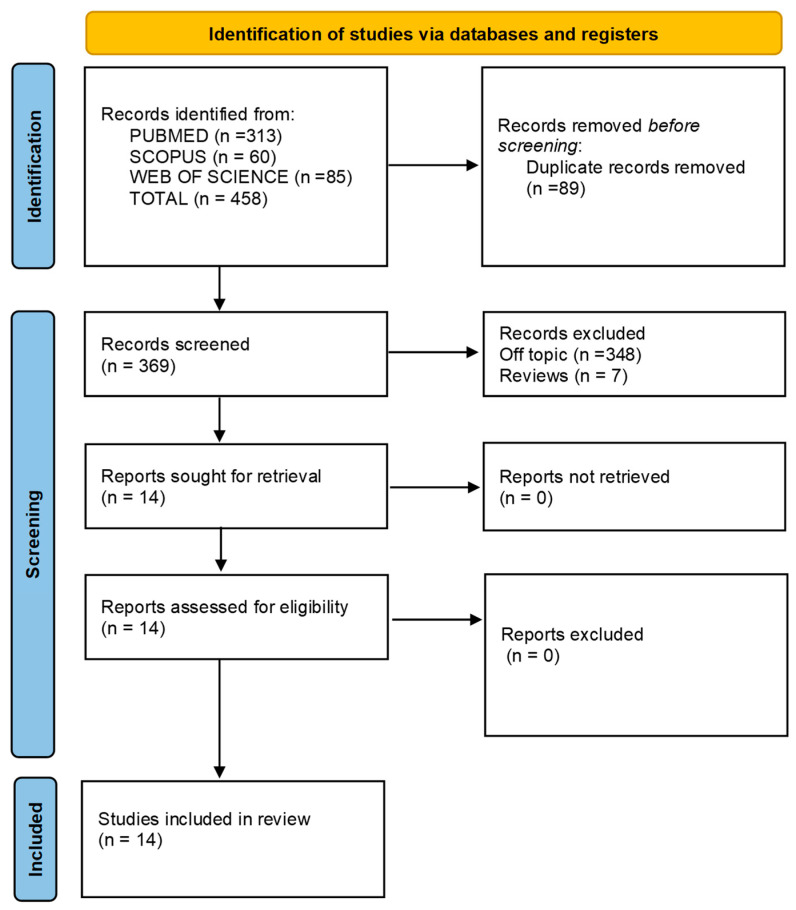
Descriptive summary of items selection.

The electronic database search yielded a total of 458 records (Scopus: *n* = 60; PubMed: *n* = 313; Web of Science: *n* = 85), with no additional articles identified through hand searching. After removing duplicates, 369 unique records were screened by title and abstract, focusing on studies involving orthodontic treatment with clear aligners and TADs. Of these, 355 were excluded for not meeting the inclusion criteria (348 were off-topic and 7 were reviews), resulting in 14 studies being included for qualitative synthesis. The selection process and an overview of the included studies are presented in Table 4 and Figure 3, respectively. It is important to note that many of the studies included in Table 4 are case reports or small case series, reflecting the novelty of combining aligners with TADs and the limited number of clinical applications currently available in the literature. The small sample sizes are primarily due to the exploratory nature of these early studies and the relatively recent integration of these two techniques in clinical practice.

### 3.2. Quality Assessment and Risk of Bias of Included Articles

The risk of bias assessment for the included studies is summarized in Table 5. Most studies showed a high risk of bias due to confounding factors. In contrast, the risk of bias related to measurement was generally low. Similarly, most studies demonstrated a low risk of bias in the selection of participants. However, bias due to post-exposure factors could not be adequately assessed because of substantial heterogeneity among the studies. The risk of bias due to missing data was low in many of the included studies. The risk of bias arising from the measurement of outcomes was also predominantly low. Conversely, a high risk of bias was noted in the selection of reported results across most studies. Overall, one study was judged to have a low risk of bias, nine studies had some concerns, four studies had a high risk of bias, and the remainder had an unclear or questionable risk of bias.

## 4. Discussion

A certain degree of heterogeneity was observed among the included studies, particularly regarding study designs, treatment protocols, and patient characteristics, which may influence the interpretation of the overall findings. Moreover, many of the included studies are limited by small sample sizes, a predominance of case reports, and a scarcity of randomized controlled trials, which collectively constrain the strength of the available evidence.

### 4.1. Integration of TADs in Clear Aligner Therapy

The involvement of TADs in clear aligner therapy represents a significant innovation in orthodontics, offering new treatment possibilities for complex malocclusions. These studies emphasize the biomechanical advantages of combining TADs with aligners, contributing to improved treatment predictability and clinical outcomes [63].

### 4.2. Applications in Class III Malocclusions and Molar Distalization

Aligner orthodontics combined with TADs have shown significant efficacy in addressing Class III malocclusions through lower molar distalization. This non-extraction approach not only improves anterior overbite but also establishes functional Class I occlusion, reducing the total number of aligner stages and minimizing patient discomfort. Tailored treatment planning is crucial in achieving these results [64,65,66].

### 4.3. Digital Orthodontics and Canine Impaction

Capuozzo et al. highlighted the refined use of digital orthodontics in planning treatments for canine impaction. Utilizing advanced imaging and simulation tools allows for precise device positioning, enhancing the effectiveness of TADs combined with aligners. This combination not only improves oral health but also increases patient compliance due to the advantages of aesthetics and comfort over traditional braces.

The new approach reinforced the efficacy in managing difficult cases [54,67,68,69,70]. In detailing a case report of clear aligner treatment with miniscrews as TADs for Class II Division 2 malocclusion accompanied by severe upper canine displacement, Wang et al. extended the clinical applicability. However, the application of miniscrews facilitates the repositioning of displaced teeth effectively and minimizes discomfort, thereby enhancing treatment effectiveness [58,71,72,73,74].

### 4.4. Addressing Alveolar Protrusion and Vertical Control

Choi et al. treated alveolar protrusion with miniscrews and clear aligners. This study highlights the shortcomings of the conventional bracket and wire procedures used in orthodontic treatments, which can be an arduous and unpleasant procedure. TADs with aligners offer accurate and consistent tooth movements, which are efficient and predictable in orthodontic treatment. This approach highlights the key role of digital orthodontic planning in personalized patient treatment [59,75,76,77,78].

### 4.5. Treatment of Anterior Open Bites

The literature, including the work of Sabouni et al., supports the use of clear aligners in treating anterior open bites. Digital orthodontic software facilitates specific tooth movements, ensuring even and progressive force distribution. Lifestyle changes are often critical to this non-invasive procedure, which yields excellent occlusion improvements in less time compared to more burdensome protocols [60,79,80,81,82].

### 4.6. Surgical Approaches in Advanced Cases

However, in more advanced cases with skeletal discrepancy, the skeletal anchorage and clear aligner approaches combined with a surgery-first protocol may effectively address the need. This was further demonstrated by Iodice et al. in the case of gummy smile and Class II with mandibular retrognathia and deviation corrections. Through the clever use of TADs, advanced imaging, and simulation software, the treatment plan in question not only improved the accuracy of the procedure but also helped increase the aesthetic and functional outcomes [61,83,84,85,86]. In a case report, Greco et al. presented the G-Block TAD system, which enables posterior skeletal anchorage after the molar distalization with aligners. This system constitutes a strong point of anchorage, enabling the efficient application of the aligners in repositioning molars, especially in adult patients with difficult orthodontic treatment. Their results present several clues regarding the possible use of TADs in conjunction with aligners [51,87,88,89,90]. Regarding impacted canines, Greco et al. used aligners and TADs to resolve such difficult cases. This methodology is therefore useful in that it is less traumatic and more comfortable than the conventional approaches, as it facilitates accurate translation of teeth whilst enhancing retention. The results they provide in their case report support the utilization of these combined techniques in such cases where aligners alone are not sufficient [52,91,92,93,94].

### 4.7. Long-Term Stability in Hyperdivergent Cases

The progress does not stop at Lu et al., who applied a plastic-defying thin scope; they preset intrusion bulbs within clear aligners for vertical control in hyper-divergent skeletal Class II malocclusion cases. Their case report, with a follow-up of four years, exemplifies the long-term stability of this technique while highlighting the effectiveness of the preformed intrusion bulbs in vertical control and treatment maintenance [53,58,95,96,97]. Park et al. discussed the integration of TADs and Invisalign in complex cases, which would be otherwise difficult to manage with large movements of teeth or accurate anchorage. As depicted in their article, TADs act as an extra apparatus in addition to double arch wires, providing a point of support that is not reliant on the teeth of the patient, thus augmenting the effectiveness of the Invisalign system and allowing many more applications of this effective aligner system [26,98,99,100,101,102].

### 4.8. Forces and Limitations of TSADs

De Almeida et al. examined the forces exerted by clear aligners associated with TSADs and discussed the possibilities and limitations of using braces and the properties of aligners as separate appliances. TADs, small temporary implants, provide additional anchorage not connected to the teeth, enabling complex movements such as large translations, rotations, and vertical adjustments. Additionally, the materials used for aligners—typically thermoplastic polymers such as polyurethane or polyethylene terephthalate glycol (PETG)—play a crucial role in treatment performance. Their flexibility, elastic recovery, and transparency affect force delivery, durability, and patient compliance. Future studies should investigate how material properties influence biomechanics when aligners are used in conjunction with TADs [62,103,104,105,106].

### 4.9. Molar Distalization and Asymmetries

Auladell et al. also examined the applicability of mini-implants in molar distalization. While clear aligners are aesthetically appealing, they often underperform in molar distalization due to reliance on intraoral anchorage. Stable anchorage through mini-implants enhances aligners’ ability to achieve accurate and effective distalization, improving treatment efficacy and reducing duration [55,107,108,109,110]. Kottemann et al. investigated the use of TADs with clear aligners for the treatment of dental asymmetries. They stressed that although the promising aesthetic and comfort features of aligners attract more and more patients, it is almost impossible to treat more complicated asymmetries because of natural anchorage. TADs make extra anchors available, which orthodontists exploit to apply and control forces in a more focused and dispersed manner, thereby improving accuracy in treatment and shortening recovery periods [56,111,112,113,114].

### 4.10. Non-Extraction Approaches in Class II Malocclusions

Ojima et al. addressed the role of clear aligners in achieving proper functional occlusion without tooth extraction in patients with Class II malocclusion. Their advancements demonstrate that excursive movements of incisors, typically requiring invasive procedures, can be achieved by combining TADs with clear aligners [39,115,116,117,118,119,120]. Last but not least, Lin et al.’s study on the three-dimensional measurement and analysis of distalization of mandibular molars achieved by micro-implant anchorage in combination with clear aligners provides crystal-clear insight into the involved and resultant forces and tooth movements [121,122,123,124,125,126,127]. Their findings emphasize a need for advanced three-dimensional analyses to optimize and assess orthodontic treatments and show how such an integrated approach can achieve more precise, effective, and predictable results [128,129,130,131,132]. The integration of TADs with advanced digital planning and clear aligner therapy has significantly impacted orthodontic practice. These combined modalities improve precision and treatment predictability while contributing to greater patient comfort and acceptance, particularly in complex cases.

### 4.11. Critical Analysis and Future Directions

Despite promising outcomes, the current evidence base is limited by methodological weaknesses, including small sample sizes, a lack of control groups, short follow-up periods, and the predominance of descriptive designs. Few randomized controlled trials exist, and heterogeneity in treatment protocols hinders direct comparison. There is a clear need for large-scale, long-term prospective studies with standardized methodologies to confirm the efficacy and predictability of aligner therapy with TADs. Future research should focus on comparative effectiveness, patient-reported outcomes, material behavior under load, and the long-term stability of results, especially in complex cases.

## 5. Conclusions

The combined use of aligners and TADs appears to be a promising approach for managing complex orthodontic cases that require significant tooth movements, such as intrusion, extrusion, and distalization. TADs provide stable anchorage, which may contribute to more controlled and predictable tooth movements, potentially improving treatment outcomes. Additionally, this combination can enhance patient satisfaction due to the aesthetic and comfort-related advantages of aligners. However, despite these encouraging findings, the current evidence must be interpreted with caution. The studies included in this review are limited in number and characterized by considerable heterogeneity in design, clinical protocols, and outcome measures. Furthermore, most of the available studies involve relatively small sample sizes, which limits the generalizability of the conclusions. There remains a clear need for high-quality randomized controlled trials to confirm the effectiveness and safety of combining TADs with aligners and to establish standardized clinical guidelines. Future research should focus on optimizing protocols, improving miniscrew placement accuracy, and defining criteria for case selection to fully harness the potential of this treatment modality.

## Figures and Tables

**Table 1:** TADs specifications, materials, and clinical indications.

Featur	Details
Material	Medical-grade titanium (Grade 5, Ti-6Al-4V)
Dimensions	Diameter: 1.2–2.0 mm; Length: 6–10 mm
Insertion Sites	Alveolar bone, palatal bone, infrazygomatic crest, interradicular areas
Insertion Technique	Manual or motor-driven, performed under local anesthesia
Placement Duration	Temporary (from a few months to completion of desired movement)
Clinical Indications	Intrusion, extrusion, molar distalization, correction of open/deep bites
Auxiliary Devices	Elastics, coil springs, direct attachments to aligners
Main Benefits	Stable anchorage, precise control of dental movements, biomechanical support
Potential Complications	Failure of integration, patient discomfort, mobility, localized infection

**Table 2:** Database search indicators.

Database	Timespan	Keywords (Boolean Operators)
PubMed, Scopus, Web of Science	1 January 2004–17 July 2024	(aligners OR clear aligners OR transparent aligners OR Invisalign) AND (Temporary Anchorage Devices OR TADs OR OrthoTADs OR Mini-implants in orthodontics OR Skeletal anchorage OR Mini-screws OR Micro-implants orthodontics OR Orthodontic anchorage systems OR Miniscrew implants OR Temporary skeletal anchorage devices OR Orthodontic biomechanics with TADs)

**Table 4:** Preferred Reporting Items for Systematic Reviews and Meta-Analyses (PRISMA) flow diagram and indicators of database search.

Authors	Study Design	Number of Patients	Average Age/Gender	Treatment and Duration	Outcomes
Greco et al. (2022) [51]	Case report	1	25 (F)	Posterior anchorage device TADs-supported after molar distalization with aligners with 15 months of treatment	Effective molar distalization and posterior anchorage using TADs and aligners
Greco et al. (2022) [52]	Case report	1	16 (M); 43 (F)	Impacted canine management using aligners supported by orthodontic TADs; treatment performed with 43 and 38 aligners, respectively	Successful alignment of impacted canine with improved patient comfort and aesthetic appeal using TADs with aligners
Lu et al. (2023) [53]	Case report	1	31 (F)	Preformed intrusion bulbs on clear aligners for vertical control in a hyperdivergent skeletal Class II case with extraction with a total of 71 sets of aligners	Improved vertical control and stability in hyperdivergent skeletal Class II case, with effective long-term results over a 4-year follow-up period
Capuozzo et al. (2023) [54]	Case series	2	17 (F), 18 (F)	Microimplant-assisted aligner therapy; treatment duration of 20 and 18 months respectively	A Class I canine bond was established, with overbite and overjet normalized. The coordination of the maxillary and mandibular midlines was completed.
Auladell et al. (2022) [55]	Case series	2	40 (M), 28 (F)	Molar distalization using clear aligners and mini-implants with 24 and 20 months of treatment, respectively	Effective molar distalization with improved treatment precision and reduced duration using mini-implants and aligners
Kottemann et al. (2020) [56]	Case series	2	37 (F), 60 (F)	Use of TADs with clear aligners for asymmetry correction with 8 and 15 months of treatment respectively	Improved asymmetry correction with precise control and efficient tooth movement using TADs and clear aligners
Lin et al. (2024) [57]	Prospective study	17	Not specified	Three-dimensional measurement and analysis of mandibular molar distalization with micro-implant anchorage and clear aligners. Group A (ten cases) received no additional anchoring, while Group B (seven cases) received micro-implant anchorage to aid in mandibular molar distalization; treatment duration not specified.	Using micro-implant anchoring and a clear aligner for mandibular molar distalization protects the central incisor and increases crown distalization efficiency.
Ojima et al. (2020) [39]	Case series	2	27 (F), 18 (M)	Aligner treatment with TADs for distalization of lower molars; 16 and 43 months of treatment, respectively	Achieved Class I molar relationship, improved facial profile, stable occlusion, significant improvement in occlusion and facial aesthetics
Wang et al. (2024) [58]	Case report	1	19 (M)	Clear aligner treatment assisted by mini screws for Class II division 2 malocclusion and right upper canine correction; treatment duration of 19 months	Corrected malocclusion, repositioned upper canine, improved dental and facial aesthetics, high patient satisfaction and compliance
Choi et al. (2009) [59]	Case report	1	16 (F)	Clear aligners and miniscrews for bialveolar protrusion; treatment duration of 21 months	Effective retraction of anterior teeth, improved aesthetics and function, patient comfort and satisfaction
Sabouni et al. (2022) [60]	Case series	3	18 (M), 25 (F), 22 (F)	Clear aligner therapy for anterior open bite; duration varies from 10 to 12 to 35 months	Significant occlusal improvements, effective closure of anterior open bites, high patient satisfaction and compliance
Iodice et al. (2021) [61].	Case report	1	21 (F)	Full digital surgery-first approach using skeletal anchorage and clear aligners for the correction of gummy smile and Class II malocclusion with mandibular retrusion and deviation; treatment duration of 15 months	Significant aesthetic and functional improvements, reduced gummy smile, corrected malocclusion, stable occlusion, harmonious facial profile
Park et al. (2020) [26]	Case series	3	30 (F), 32 (M), 41 (M)	Use of TADs with Invisalign to facilitate difficult tooth movements, treatment duration specified only for the first case report of 34 sets of aligners	Successful management of difficult orthodontic movements, with enhanced precision and control using TADs with Invisalign
de Almeida et al. (2024) [62]	Case series	3	12 (M), 15 (M), 20 (M)	Biomechanics of clear aligners associated with TADs with 26, 11, and 15 months of treatment	Enhanced efficiency and predictability of complex tooth movements with the integration of TSADs and clear aligners

**Table 5:** Bias assessment.

Authors	D1	D2	D3	D4	D5	D6	D7	Overall
Ojima et al. (2020) [39]	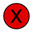	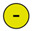	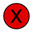	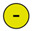	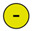	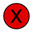	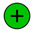	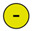
Capuozzo et al. (2023) [54]	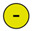	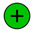	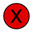	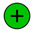	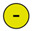	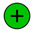	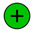	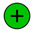
Wang et al. (2024) [58]	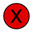	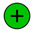	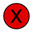	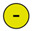	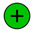	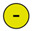	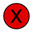	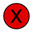
Choi et al. (2009) [59]	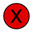	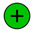	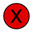	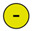	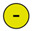	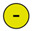	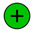	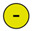
Sabouni et al. (2022) [60]	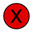	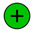	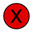	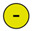	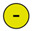	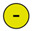	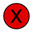	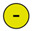
Iodice et al. (2021) [61]	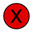	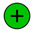	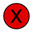	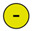	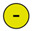	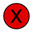	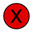	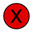
Greco et al. (2022) [51]	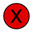	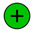	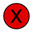	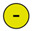	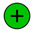	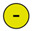	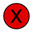	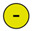
Greco et al. (2022) [52]	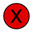	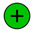	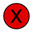	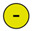	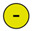	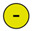	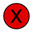	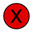
Lu et al. (2023) [53]	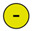	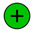	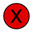	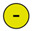	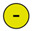	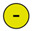	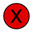	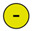
Park et al. (2020) [26]	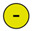	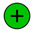	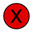	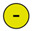	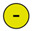	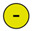	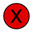	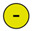
de Almeida et al. (2024) [62]	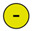	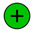	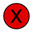	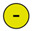	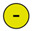	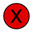	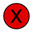	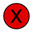
Auladell et al. (2022) [55]	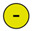	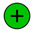	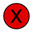	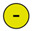	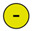	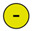	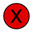	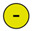
Kottemann et al. (2020) [56]	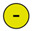	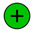	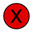	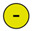	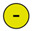	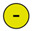	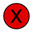	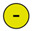
Lin et al. (2024) [57]	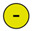	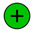	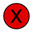	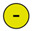	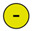	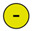	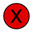	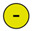

Domains: D1: Bias due to confounding. D2: Bias arising from the measurement of the exposure. D3: Bias in the selection of participants in the study (or in the analysis). D4: Bias due to post-exposure interventions. D5: Bias due to missing data. D6: Bias arising from the measurement of the outcome. D7: Bias in selection of the reported result. 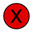 High. 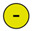 Some concerns. 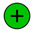 Low.

## Abbreviations

The following abbreviations are used in this manuscript:

TADs	temporary anchorage devices
